# Re-evaluation of pulmonary titanium dioxide nanoparticle distribution using the "relative deposition index": Evidence for clearance through microvasculature

**DOI:** 10.1186/1743-8977-4-7

**Published:** 2007-08-29

**Authors:** Christian Mühlfeld, Marianne Geiser, Nadine Kapp, Peter Gehr, Barbara Rothen-Rutishauser

**Affiliations:** 1 Institute of Anatomy, University of Bern, Baltzerstrasse 2, CH-3000 Bern 9, Switzerland

## Abstract

**Background:**

Translocation of nanoparticles (NP) from the pulmonary airways into other pulmonary compartments or the systemic circulation is controversially discussed in the literature. In a previous study it was shown that titanium dioxide (TiO_2_) NP were "distributed in four lung compartments (air-filled spaces, epithelium/endothelium, connective tissue, capillary lumen) in correlation with compartment size". It was concluded that particles can move freely between these tissue compartments. To analyze whether the distribution of TiO_2 _NP in the lungs is really random or shows a preferential targeting we applied a newly developed method for comparing NP distributions.

**Methods:**

Rat lungs exposed to an aerosol containing TiO_2 _NP were prepared for light and electron microscopy at 1 h and at 24 h after exposure. Numbers of TiO_2 _NP associated with each compartment were counted using energy filtering transmission electron microscopy. Compartment size was estimated by unbiased stereology from systematically sampled light micrographs. Numbers of particles were related to compartment size using a relative deposition index and chi-squared analysis.

**Results:**

Nanoparticle distribution within the four compartments was not random at 1 h or at 24 h after exposure. At 1 h the connective tissue was the preferential target of the particles. At 24 h the NP were preferentially located in the capillary lumen.

**Conclusion:**

We conclude that TiO_2 _NP do not move freely between pulmonary tissue compartments, although they can pass from one compartment to another with relative ease. The residence time of NP in each tissue compartment of the respiratory system depends on the compartment and the time after exposure. It is suggested that a small fraction of TiO_2 _NP are rapidly transported from the airway lumen to the connective tissue and subsequently released into the systemic circulation.

## Background

A growing number of epidemiological studies suggests that airborne particles convey adverse health effects in humans causing increased morbidity and mortality [1-3]. Experimental work has provided evidence for the generation of reactive oxygen species as well as inflammatory and genotoxic responses upon exposure to particulate matter both in animal models and in vitro studies [4-8]. In recent years special emphasis has been placed on particles smaller than 100 nm at least in one dimension [9]. These are usually referred to as ultrafine particles if they have their origin in gas-to-particle conversion or incomplete combustion processes or as nanoparticles (NP) if they are manufactured synthetically. In the following, we use the term NP independent of particle origin or shape.

The growing interest of toxicologists in NP is mainly due to the following reasons: First, the obvious advances made in nanotechnology are accompanied by a lack of knowledge about the potential health risks of NP [9]. Second, while nanotechnology exploits the fact that NP may exhibit different biophysicochemical characteristics than particles of the same material at a larger scale [10], these differences may also hold true for the interaction of NP with biological systems [11]. In consequence, the production of NP, even if composed of materials formerly not known to be toxic, and the subsequent occurrence of NP in water, soil or air will sooner or later bring about an exposition of humans to NP. Due to the large size of the alveolar surface (approximately 140 m^2 ^in the human) and the minimal distance between air and blood (approximately 0.2 – 0.4 μm in the thin regions), the lungs are an ideal portal of entry for airborne NP [12].

**Figure 1:**
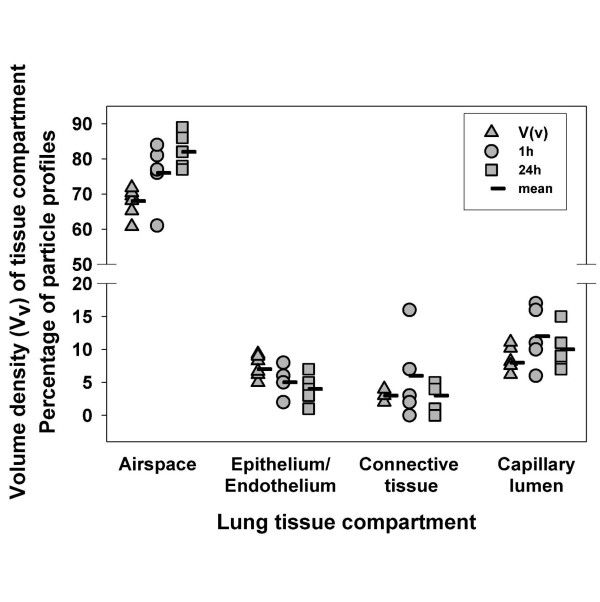
Original data from Geiser et al. [18] showing the mean number of TiO_2 _NP in the four defined tissue compartments at 1 h and at 24 h after exposure. Volume fractions of the compartments are also shown. Reproduced from Geiser et al. [18]. With permission.

One intriguing question relates to the translocation characteristics of NP. Despite a growing body of literature, there is still a controversial debate whether NP are able to cross the pulmonary blood-air barrier and, hence, are translocated to the blood circulation in a significant amount [13,14]. Interestingly, animal and in vitro studies provide evidence for such translocation properties of NP [15,16] whereas most investigations in the human show a very limited translocation to the circulation if any [14,17]. In fact, in a recent study we were able to show that titanium dioxide (TiO_2_) NP are able to cross cellular membranes in a rat lung exposure model that did not involve commonly known phagocytotic mechanisms [18]. This study raised the possibility of TiO_2 _NP being able to enter cells and tissues in a rather unrestricted fashion. A corollary of such particle characteristics would be a random particle distribution, i.e. the number of particles observed within a certain compartment should be correlated to the size of the compartment. Indeed, Geiser et al. [18] provided a Figure (Figure 1) that related the volume fractions of pulmonary compartments to the number of counted particles. At that time, it was concluded from the correlation between volume fractions and particle numbers that "particles can move between tissue compartments without restraint" [18].

Very recently, however, a new method was developed to analyse the distributions of NP within tissues, cells or cell organelles in more detail [19]. The new method basically relates the size of a tissue (or intracellular) compartment to the number of particles located within a particular compartment. From these data, a relative deposition index (RDI) can be calculated which indicates whether a compartment contains more (RDI > 1), the same (RDI = 1) or less (RDI < 1) particles than would be expected from its size. Using the chi-squared test, we can further test the null hypothesis that the distribution of NP among the tissue compartments is random and if not, identify those compartments which contribute substantially to this difference. Those compartments with an RDI > 1 and a substantial contribution to the non-randomness of particle localization are identified as preferential targets of the particles.

As this method is very sensitive and reliable for analysing the distribution of particles in relation to compartment size the present study investigates whether the particle distribution reported by Geiser et al. [18] is really random or shows a preferential localization of the particles. The conclusions that can be drawn from this re-evaluation are important for understanding particle translocation characteristics because we show that particle distributions are neither random at 1 h or at 24 h after exposure. This result contradicts the former concept of an unrestricted NP movement [18] and rather points to controlled translocation processes.

## Results and Discussion

**Figure 2:**
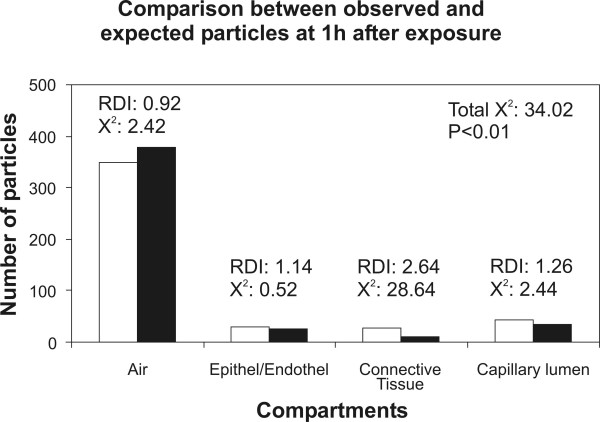
Illustration of the observed (white columns) and the expected (black columns) NP within the four tissue compartments at 1 h after exposure. The total chi-squared showed that the distributions of observed and expected particles differed significantly. There is only one compartment with an RDI > 1 and a substantial contribution to the total chi-squared: the connective tissue is the only compartment that is preferentially targeted by the NP at 1 h after exposure.

**Figure 3:**
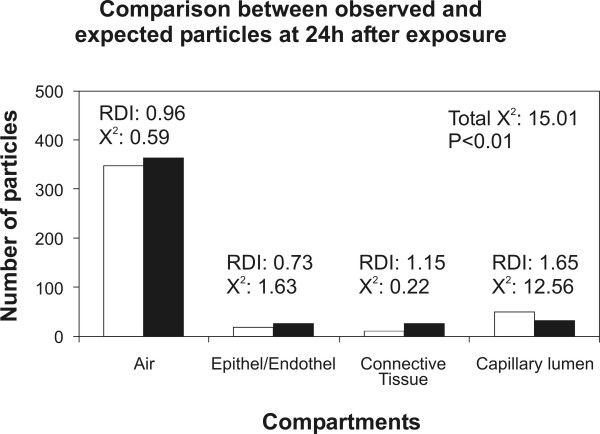
Illustration of the observed (white columns) and the expected (black columns) NP within the four tissue compartments at 24 h after exposure. The total chi-squared showed that the distributions of observed and expected particles differed significantly. There is only one compartment with an RDI > 1 and a substantial contribution to the total chi-squared: the capillary lumen is the only compartment that is preferentially targeted at 24 h after exposure.

In the present study we re-evaluated the distribution of titanium dioxide NP in lung tissue of rats exposed to an aerosol containing NP for 1 h. The tissue distribution of NP was analyzed at 1 h and at 24 h after exposure. The rationale for the re-evaluation was that the original report [18] had already related the number of NP associated with each compartment to the compartment size (Fig. 1) but a sound tool to compare these data statistically was lacking. Very recently, some of the authors have adopted a quantitative method for analyzing the intracellular distribution of immunogold particles [20,21] to NP research. This method was originally introduced using hypothetical sets of data [19] and allows the analysis of NP distributions in tissue and cellular compartments in a very sensitive and statistically valid way. The results of this analysis are reported in Tables 1 and 2 and in Figures 2 and 3.

**Table 1:** Analysis of TiO_2_-nanoparticle distribution 1 h after exposure.

**Compartment**	**N_O_**	**P**	**N_E_**	**RDI**	**Χ^2^**	**Fraction of total Χ^2^**
**Air**	348	2251	378	0.920	2.42	0.071
**Epi/Endo**	29	151	25	1.143	0.52	0.015
**CT**	28	63	11	2.645	28.64	0.842
**Caplum**	44	207	35	1.265	2.44	0.072
**Total**	449	2672	449		34.02	

Note. From the observed number of particles (N_O_) and the number of points (P) on each compartment the expected number of particles (N_E_) was calculated (e.g. N_E _(air) = 449 × (2251/2672) = 378). The RDI is calculated from N_O_/N_E _(e.g. RDI (air) = 348/378 = 0.92). The chi-squared (Χ^2^) values are calculated from (N_O _- N_E_)^2^/N_E _(e.g. Χ^2 ^(air) = (348–378)^2^/378 = 2.42). With three degrees of freedom (2-1 groups × 4-1 compartments) and a total chi-squared value of 34.02, the null-hypothesis of random distribution has to be rejected (p < 0.01). The connective tissue has an RDI of 2.645 and a partial chi-squared value that contributes about 84% of the total chi-squared. It is the only compartment that meets both criteria for a preferential deposition. Abbreviations: Air: Air space; Epi/Endo: Epithelial/endothelial cells; CT: Connective tissue; Caplum: Capillary lumen. Χ^2^: Chi-squared test values. N_O_: Observed number of particles. N_E_: Expected number of particles. RDI: Relative deposition index.

**Table 2:** Analysis of TiO_2_-nanoparticle distribution 24 h after exposure.

**Compartment**	**N_O_**	**P**	**N_E_**	**RDI**	**Χ^2^**	**Fraction of total Χ^2^**
**Air**	348	2052	363	0.96	0.59	0.039
**Epi/Endo**	17	131	23	0.73	1.63	0.109
**CT**	11	54	10	1.15	0.22	0.015
**Caplum**	49	168	30	1.65	12.56	0.837
**Total**	425	2405	425		15.01	

Note. The calculation of the data is described in the Methods section and an illustrating example is provided in the note to table 1. With three degrees of freedom (2-1 groups × 4-1 compartments) and a total chi-squared value of 15.01, the null-hypothesis of random distribution has to be rejected (p < 0.01). The capillary lumen has an RDI of 1.65 and a partial chi-squared value that contributes about 84% of the total chi-squared. It is the only compartment that meets both criteria for a preferential deposition. Abbreviations: Air: Air space; Epi/Endo: Epithelial/endothelial cells; CT: Connective tissue; Caplum: Capillary lumen. Χ^2^: Chi-squared test values. N_O_: Observed number of particles. N_E_: Expected number of particles. RDI: Relative deposition index.

In contrast to the conclusions drawn from our previous report, the present study shows that the distribution of NP within the four defined pulmonary tissue compartments is neither random after 1 h nor after 24 h after exposure. The null hypotheses tested in the analyses were: "The observed and expected NP distributions are equal." With three degrees of freedom (4-1 compartments × 2-1 groups) the null hypothesis of random distribution had to be rejected with p < 0.01 in both cases. This result indicates that despite the seeming correlation seen in Fig. 1, there is a statistically significant difference between the observed NP distribution and that expected from compartment size. With the definition that a preferential compartment localization of the particles has to meet two criteria, namely RDI > 1 and partial chi-squared/total chi-squared > 10%, we can identify the connective tissue as the preferential target of NP at 1 h after exposure and the capillary lumen as the preferential target at 24 h after exposure. Therefore, NP distribution within the four compartments is not only non-random but there is also a change in the preferential localization due to time after exposure. We found a preferential localization in the connective tissue at 1 h and in the capillary lumen at 24 h after exposure.

Although it is not possible to provide a clear mechanistic image of the translocation characteristics of TiO_2 _NP from these data alone there are several conclusions that can be drawn from the results. First of all, the data do not support the hypothesis that NP can travel between different pulmonary compartments without restraint. In such case, all compartments should have had an RDI of approximately 1 and the total chi-squared would not have reached the significance level [19]. Second, NP seem to be transported from the airspaces to the connective tissue rapidly after exposure which includes that the epithelium is rather a transit passage for NP than a preferential target. However, a third conclusion is that NP are not transported to the connective tissue for being deposited for long time storage since they do not contribute substantially to the non-randomness of the distribution at 24 h after exposure. In fact, after 24 h the preferential target of the NP is the capillary lumen which indicates that NP are shifted from the connective tissue into the circulation where they are retained. It needs to be mentioned at this point that the counting of the particles inside the capillary lumen raises several issues. The capillary lumen and, hence, the blood is a non-static compartment, therefore the number of particles inside the circulation may be expected to be higher than the number counted. However, NP were observed in the free lumen, mostly near but not in direct contact to the capillary endothelium. Upon additional screening, very few particles were also observed inside erythrocytes left in the capillaries. Therefore, due to the non-static nature of the circulation and the mode of fixation (perfusion fixation) the number of NP inside the capillaries was possibly underestimated in the present study.

How do these results and conclusions fit into the current knowledge about NP translocation from the airspaces to the blood circulation? It is well known that NP, in contrast to larger sized particles, reach the alveolar region of the lung and remain there for a much longer period of time after exposure [22]. Furthermore, Oberdörster et al. [22] showed that a large fraction of the retained NP was present in the interstitial space but decreased after a certain period of time after exposure. In a more recent study, Takenaka et al. [23] studied the translocation of ultrafine gold particles in rat lungs during several days after a 6 h inhalation exposure. They found that the gold particles were taken up by alveolar macrophages and epithelial cells and were also present in the blood circulation. Kreyling et al. [24] showed a translocation of iridium NP from the lungs to extrapulmonary organs and the circulation which was very low. Nemmar et al. [13] provided evidence for a significant translocation of inhaled carbon NP to the blood circulation, however, when they used polystyrene particles in an isolated rabbit lung model they did not observe such translocation [25]. The concept of a significant clearance of NP by translocation to the circulation has been challenged by two recent studies (similarly designed as [13]) on inhaled carbonaceous particles [14,17]. These authors did not find evidence for a significant clearance of NP retained in the human lung, especially not by the blood circulation.

In light of these studies, our analysis supports the concept of pulmonary clearance of TiO_2 _NP through the microvasculature, however, the RDI must not be misunderstood with respect to the total number of NP. In relation to the total number of NP within the lung the number of TiO_2 _NP observed within the capillaries is still very low (Tables 1 and 2). Other routes of clearance (exhalation, mucociliary escalator) which may decrease the number of NP would certainly decrease the total number of observed NP and therefore increase the RDI for the other compartments. Nonetheless, clearance from the airspaces via the bronchial tree seems to be very low and NP are retained in the lungs for a long period of time [22,23] and particles were still present in the airspaces to a similar extent in the rat lungs at 24 h after exposure compared with those at 1 h after exposure. It is beyond the scope of our results to evaluate whether this translocation to the circulation has functional significance and may cause adverse health effects. However, the data do support that the circulation is a preferential target of retained NP. Obviously, the comparison between the many different studies on particle translocation is hampered by the application of different types of species and NP, exposure models and durations, the duration between exposure and analysis and other factors. The different physicochemical properties of the NP applied in the studies may be the most influential factor and may seriously influence the translocation characteristics. In this regard, it has been recognized that there is a need to standardize several experimental procedures in order to make studies comparable [9]. However, the present study also underlines the necessity of expressing NP distributions in quantitative terms and in relation to compartment size. We believe that this new quantitative microscopic method – if regularly applied – will help to enhance the comparability between studies where NP distribution is relevant because it combines the high resolution of transmission electron microscopy with a reliable statistical tool. It will also enhance the correlation between morphological observation and functional results.

## Conclusion

The present study contributes to the understanding of nanoparticle translocation characteristics in the lung. It showed that the distribution of inhaled titanium dioxide particles in the rat lung was not random, thus pointing at a regulated translocation process rather than a diffusive particle movement. The connective tissue was the preferential target of the studied particles at 1 h after exposure. A significant translocation and retention of the particles took place during the subsequent period, making the capillary lumen the preferential target at 24 h after exposure. The present study also stresses the need and usefulness of appropriate quantitative methods for the study of nanoparticle distributions.

## Methods

### Experiments

The experiments investigated in the present study were described in detail previously [18]. Important methodological features of the experiments are given in Table 3.

**Table 3:** Important methodological issues of the experiments. For details see Geiser et al. [18].

**Animals**	
Species	Adult male WKY/NCrl BR rats
Number	n = 5 for each group
**Particles**	
Material	Titanium dioxide
Aerosol generation	Spark generator (Palas) in a pure argon plus 0.1% oxygen stream
Count median diameter (nm)	22 (SD 1.7)
Exposure	Inhalation of aerosol for 1 h

**Fixation and tissue processing**	
Time point of fixation	1 h or 24 h after particle exposure
Fixation mode	Subsequent perfusion fixation with 2.5% buffered glutaraldehyde, 1% osmium tetroxide, 0.5% uranyl acetate
Tissue sampling	Systematic uniform random sampling
Material for light and electron microscopy	Semithin (toluidine staining) and ultrathin (uranyl acetate and lead citrate staining) sections

### Particle number and compartment size

From the qualitative investigation, four major compartments had been defined in which the TiO_2 _particles were found: air-filled spaces, connective tissue, epithelium/endothelium, capillary lumen. Titanium dioxide NP numbers associated with each compartment were taken from the original data published by Geiser et al. [18]. Briefly, the original data were gathered by counting particle profiles in systematic uniform random test fields of ultrathin tissue sections using a LEO 912 transmission electron microscope (Zeiss, Oberkochen, Germany). Particle identity was verified by element analysis (energy filtering transmission electron microscopy) as described in detail previously [26,27].

In the original report, information about compartment size was taken from older studies on rat myocardium [18]. For this study, compartment size was estimated from the same lungs that were used for counting NP numbers. In short, semithin sections of pulmonary tissue were sampled in a systematic uniform random fashion [28,29] using an Axioskope light microscope (Zeiss, Oberkochen, Germany) equipped with a computer assisted stereology tool (CAST-Grid 2.0, Olympus, Denmark). On each test field, a point grid was superimposed and points hitting the defined compartments were counted. The number of points hitting each compartment is an unbiased measure for the size (volume) of each of the compartments [30].

Since the total numbers of particles and points counted for each animal were different and every animal should make the same contribution to the further analysis, the raw counts were converted to normalized point and particle counts by multiplying the total counts with the mean fraction of each compartment. Data of raw and normalized particle and point counts are given in Table 4.

**Table 4:** Number of observed points and particles

	**Air**	**Epi/Endo**	**CT**	**Caplum**	**Total**
**P (1 h)**	2250 ± 396	153 ± 46	63 ± 10	206 ± 51	2672 ± 478
**P (24 h)**	2058 ± 445	128 ± 12	54 ± 12	165 ± 19	2405 ± 470
**Normalized P (1 h)**	2251 ± 62	151 ± 30	63 ± 9	207 ± 43	2672
**Normalized P (24 h)**	2052 ± 42	131 ± 19	54 ± 4	168 ± 28	2405
**N_O _(1 h)**	343 ± 218	35 ± 40	33 ± 38	38 ± 20	449 ± 283
**N_O _(24 h)**	354 ± 193	15 ± 9	14 ± 15	44 ± 12	426 ± 218
**Normalized N_O _(1 h)**	348 ± 45	29 ± 27	28 ± 27	44 ± 20	449
**Normalized N_O _(24 h)**	348 ± 25	17 ± 13	11 ± 9	49 ± 20	426

Note. Air: Air space; Epi/Endo: Epithelial/endothelial cells; CT: Connective tissue; Caplum: Capillary lumen; N_O_: Number of observed particles; P: Number of points.

### Relative deposition index and chi-squared analysis

The normalized data entered a statistical analysis based on the comparison between observed and expected particle distributions using the chi-squared (Χ^2^) test [19]. The observed distribution of particles is given by the number of particles associated with each compartment. The expected distribution of particles is based on the hypothetical assumption of random particle distribution. If particles were expected to be distributed randomly within the pulmonary compartments then the number of particles associated with each compartment would correlate with the compartment size which is given by the observed number of points.

Therefore, the expected number of particles for each compartment (N_E _(comp)) can be calculated from the total number of observed particles (N_O _(total)) and the numbers of observed points in total (P (total)) and for each compartment (P (comp)):

N_E _(comp) = N_O _(total) × (P(comp)/P (total))

The relative deposition index (RDI) indicates whether the number of observed particles is higher (RDI > 1) or lower (RDI < 1) than the expected number. If RDI = 1, the observed and expected numbers of particles are equal. The RDI is given by:

RDI = N_O _(comp)/N_E _(comp)

with N_O _(comp) being the number of particles counted within one of the compartments.

A convenient way to compare observed and expected distributions is the chi-squared test [31]. Here, it was used to compare the observed and expected NP distributions or, in other words, to test the null hypothesis of random particle distribution. Partial chi-squared values (partial Χ^2^) are calculated as follows:

partial Χ^2 ^= (N_O_-N_E_)^2^/N_E_

The total chi-squared value is calculated by adding up all partial chi-squared values. It indicates whether the observed and expected distributions are equal, i.e. whether the null hypothesis of random particle localization can be accepted. If this is not the case, we can identify compartments of preferential particle localization if two criteria are met: the RDI is larger than 1 and the partial chi-squared value of this compartment contributes substantially (a convenient value is 10% or more) to the total chi-squared. A worked example is given in the footnote to Table 1.

## Abbreviations

NP- Nanoparticle(s).

TiO_2_- Titanium dioxide.

RDI - Relative deposition index.

N_E _(comp)- Number of expected particles for a particular compartment.

N_O _(comp)- Number of observed particles for a particular compartment.

N_O _(total)- Total number of observed particles.

P (comp)- Number of points hitting a particular compartment.

P (total)- Total number of points.

## Competing interests

The author(s) declare that they have no competing interests.

## Authors' contributions

All of the authors participated in the planning, analysis and interpretation of the experiments. CM, PG and BR developed the methodology for nanoparticle distribution analysis and carried out the re-evaluation of the data. MG and NK performed the EM analysis including counting of particles and estimation of compartment size. All authors read and approved the final manuscript.
